# Global Radiomic Features from Mammography for Predicting Difficult-To-Interpret Normal Cases

**DOI:** 10.1007/s10278-023-00836-7

**Published:** 2023-05-30

**Authors:** Somphone Siviengphanom, Ziba Gandomkar, Sarah J. Lewis, Patrick C. Brennan

**Affiliations:** Medical Image Optimisation and Perception Group, Discipline of Medical Imaging Science, Sydney School of Health Sciences, Faculty of Medicine and Health, the University of Sydney, Sydney, NSW 2006 Australia

**Keywords:** Radiomics, Mammography, Difficult normal cases, Breast cancer, Machine learning, Gist

## Abstract

This work aimed to investigate whether global radiomic features (GRFs) from mammograms can predict *difficult-to-interpret* normal cases (NCs). Assessments from 537 readers interpreting 239 normal mammograms were used to categorise cases as 120 *difficult-to-interpret* and 119 *easy-to-interpret* based on cases having the highest and lowest difficulty scores, respectively. Using lattice- and squared-based approaches, 34 handcrafted GRFs per image were extracted and normalised. Three classifiers were constructed: (i) *CC* and (ii) *MLO* using the GRFs from corresponding craniocaudal and mediolateral oblique images only, based on the random forest technique for distinguishing *difficult-* from *easy-to-interpret* NCs, and (iii) *CC* + *MLO* using the median predictive scores from both *CC* and *MLO* models. Useful GRFs for the *CC* and *MLO* models were recognised using a scree test. The *CC* and *MLO* models were trained and validated using the leave-one-out-cross-validation. The models’ performances were assessed by the AUC and compared using the DeLong test. A Kruskal–Wallis test was used to examine if the 34 GRFs differed between *difficult-* and *easy-to-interpret* NCs and if difficulty level based on the traditional breast density (BD) categories differed among 115 *low-BD* and 124 *high*-*BD* NCs. The *CC* + *MLO* model achieved higher performance (0.71 AUC) than the individual *CC* and *MLO* model alone (0.66 each), but statistically non-significant difference was found (all *p* > *0.05*). Six GRFs were identified to be valuable in describing *difficult-to-interpret* NCs. Twenty features, when compared between *difficult-* and *easy-to-interpret* NCs, differed significantly (*p* < 0.05). No statistically significant difference was observed in difficulty between *low-* and *high-BD* NCs (*p* = 0.709). GRF mammographic analysis can predict *difficult-to-interpret* NCs.

## Introduction

Among women globally, breast cancer is the most prevalent cancer type and leading cause of cancer-related death [1]. While mammography screening has played a major role in the early detection of breast cancer for decades [2–4], mammographic image interpretation is an extremely complex process with reported false-positive (FP) error rates of up to 16% and estimated cumulative risk of FP results of up to 63% in women 50–69 years of age after ten biennial mammographic screening rounds [5, 6]. A study reported that 80% of women having abnormal mammographic findings at screening were recalled for further diagnostic procedures that eventually resolved with normal outcomes and 40% of those went through invasive biopsy which subsequently followed in benign findings [7]. These unnecessary recalls (i.e., FP findings) have placed huge burdens on patients and healthcare systems, causing considerable psychological distress and anxiety as well as unwarranted medical costs (e.g., USD$2.8 billion each year in the USA) [8–11]. Considering that approximately 99.5% of cases in screening programs are normal, the ability of radiologists to interpret and detect normal cases (NCs) correctly is extremely important [12, 13].

Educational test sets are powerful web-based platforms shown to improve radiologists’ performance in interpreting mammograms [14–22]. Since NCs often constitute almost 70% of the cases in these educational test sets, the capability to identify normal mammographic features can be well assessed [19, 20, 23]. However, a consistent difficulty for normal mammographic cases across different test sets is important in order to facilitate a reliable and efficient objective measurement of FP errors and the performance of individual reader, since test set based education is widely used worldwide as a means of monitoring clinical performance [16–20, 22–25]. This implies that if a radiologist’s performance changes over time, it should be a function of the radiologist, not the test set conditions. Considering readers’ performance in educational test sets can largely signify their clinical performance; measured by clinical audits, a consistent difficulty of NCs in test set based education is also vital for clinical screening programs. It can not only enable uniform empirical measurement of the screening program’s recall rate performance over time, but also facilitate the establishment of national standards of recall rate that could be used to compare with the recall rate performance of individual breast reader as part of a quality improvement [15, 20, 22–25].

To date, there appears to be no reliable method of standardising test set difficulty for NCs since test set generation currently relies on experts’ assessment of case difficulty, where suitable NCs are selected manually by expert radiologists, often based on the traditional breast imaging and reporting data system (BI-RADS) breast density (BD) category and inclusion of benign features [18, 26–28]. Studies, however, have revealed that the difficulty of NCs subjectively defined by experts or perceived by individuals based on the BD classification rarely aligns to the actual difficulty based on readers’ performance in the test set [28–30]. Although a retrospective assessment of normal case difficulty is performed by calculating the numbers of errors made by readers engaging with the test set, this is rare, and indeed when it occurs, it relies on the efficacy of the readers of that test set which is still not a good descriptor of the actual difficulty of NCs [18, 26, 28, 29]. Consequently, there is an urgent need for an alternative standardised method of predicting NCs’ difficulty so that readers’ performance and individual specific FP errors can be better monitored, identified, addressed, and improved.

This study aims to provide a reliable method for automatically describing the difficulty of NCs (i.e., an objective measure of difficulty based on readers’ error rate [31]) using global image features [32] derived from radiomics (extracting and analysing a large number of quantitative features from medical images and converting them into multi-dimensional data) [33–38]. Radiomics in mammography, to date, has focused on predicting readers’ error making patterns using local radiomic features of specific tumour/FP areas, achieving up to 0.61 AUC [39, 40]. Previous studies have also shown that radiologists detect the gist of “abnormal” based on overall image appearance immediately after image onset [41–43]. Considering the importance of the initial global impression in guiding a visual search and making a diagnostic decision [41–45] as well as the value of radiomics in mammography [38], we hypothesised that global image statistics (i.e., global radiomic features/GRFs) of *difficult-to-interpret* NCs differed from *easy-to-interpret* ones. However, little attempt has been made to predict *difficult-to-interpret* NCs based on GRFs. This study addresses this deficiency. Moreover, since test set curation often depends on the traditional BI-RADS BD classification and higher BD cases have previously been reported to be more difficult to interpret [28, 30, 46], this work also investigates if there is a difference in difficulty level between *low-BD* (BI-RADS BD category A = fatty and B = fibroglandular) vs *high-BD* (BI-RADS BD category C = heterogeneous and D = extremely dense) NCs [47].

## Materials and Methods

Ethical approval was obtained for this study from the Human Research Ethics Committee of the University of Sydney [2019/013 and 2017/028] with informed consent acquired from each reader. This study only involved retrospective analysis of existing and non-identifiable data collected as part of the BreastScreen Reader Assessment Strategy (BREAST) databank (https://breast-australia.sydney.edu.au/) [48, 49], which is funded by the Commonwealth of Australia to improve observer performance in BreastScreen Australia.

### Readers

A total of 537 readers were included in this study, with expert breast Australian and New Zealand radiologists (*n* = 382), breast physicians (*n* = 18, medical practitioners specializing in interpreting breast images such as mammograms, and diagnosis and management of benign and malignant breast disease, in partnership with breast surgeons, radiologists, oncologists, pathologists and geneticists), and radiology trainees (*n* = 137) who engaged with the BREAST program from 04 September 2014 to 17 March 2021. These readers were recruited either through a radiology and/or breast cancer workshop/conference or a direct registration via the online BREAST platform. Additionally, radiologists may be referred to complete the BREAST test set by their lead BreastScreen Australia radiologist. The number of readers who read each test set varied from 33 to 206, in accordance with the time release of the test sets between 2014 to 2021. There was a mean value of 130 readers per test set, and each reader had an average of 10 years’ experience in reading mammograms. Out of 537, 471 readers provided their age details, giving a mean age of 46. While the number of female (44%) and male (41%) readers (15% did not specify their gender) and the number of readers who spent less than 4 h (50%) and 4–30 h per week (48%) reading mammograms were similar, 46% of readers read 20–200 cases of mammograms per week, 26% completed a fellowship which lasted for 3–6 months, and 42% currently read for breast screening program (Table 1).

**Table 1:** Readers’ details at the time of completing a test set

Total no. of readers	537
Mean no. of years reading mammograms	10
Mean age (from 471 readers with age details while 66 readers did not provide their age details)	46
Gender	
Female	238 (44%)
Male	219 (41%)
Not specified	80 (15%)
No. of mammographic cases reading per week	
< 20	230 (43%)
20–200	248 (46%)
> 200	59 (11%)
No. of hours per week reading mammograms	
< 4	267 (50%)
4–30	258 (48%)
> 30	12 (2%)
No. of readers completed a fellowship lasting 3 to 6 months	
Completed fellowship	138 (26%)
Did not completed fellowship	386 (72%)
Not specified	2 (13%)
No. of readers currently read for breast screening program	
Yes	223 (42%)
No	314 (58%)

### Normal Cases and Reading Conditions

Given the aim of the study is to predict the difficulty of NCs using GRFs, only NCs were included.NCs came from a screening population; hence, patients were all females, aged between 50 to 75, and generally asymptomatic. Truth for each normal case was validated by the consensus reading of at least two expert radiologists, followed by a subsequent negative screen outcome confirmed by two expert radiologists [14]. A total of 361 normal mammography cases from all the nine existing BREAST test sets were used, with each case including bilateral craniocaudal (CC) and mediolateral oblique (MLO) views—a total of four DICOM images per case. The NCs of each test set were viewed by the 537 readers via the BREAST platform, either at a workshop/conference in a simulated reading room (*n* = 320), or online at their usual clinical setting (*n* = 217). Reading conditions in both settings were comparable with DICOM images being displayed on a pair of 5-megapixel medical standard monitors and recommended ambient light levels range of 20–40 lux [17, 19, 48, 50, 51]. The mammogram examinations were performed using mammography provided by Hologic (Hologic, Inc., Marlborough, MA, USA), GE (GE Healthcare, Chicago, IL, USA), Siemens (Munich, Germany), Sectra (Sectra, Linköping, Sweden), Philips (Philips Healthcare, Amsterdam, the Netherlands), and Fujifilm (Fujifilm Corporation, Minato City, Tokyo, Japan).

### Difficult Normal Cases and Their Breast Density

To identify the difficulty of NCs, difficulty scores (i.e., percentage of incorrect reports per case defined as an objective measure of difficulty [31]) were firstly calculated for each of the 361 NCs using the Royal Australian and New Zealand College of Radiologists (RANZCR) rating (scale of 2 to 5 while 1 represents a normal case [48]) provided by each of the 537 readers. Similar to the American College of Radiology’s numbered BI-RADS mammographic assessment categories, a RANZCR score of 2 was given when readers thought the case was benign lesion, while a score of 3, 4, or 5 was given when readers believed a malignant lesion was present with greater number indicating a higher confidence of malignancy. For cases with more than one rating from the same reader (this happened when a reader believed there were multiple abnormalities), only the highest rating was used. Difficulty scores were derived by dividing the number of readers who gave an incorrect report (i.e., rated a normal case as cancer with a rating of 3, 4, or 5) by the number of total readers who read the test set. The difficulty scores were then categorised into three classes, giving 120 *difficult-to-interpret*, 122 *intermediate-to-interpret*, and 119 *easy-to-interpret* NCs based on one-third of the total 361 cases having the highest, intermediate, and lowest difficulty scores, respectively. However, only cases (total of 239) in the *difficult-* (*n* = 120) and *easy-to-interpret* (*n* = 119) categories were included for the analysis. The 122 intermediate NCs were excluded to enable a clear distinction between the highest and lowest difficulty NCs. These two categories also resulted in a total of 115 *low-BD* (58 *difficult-* and 57 *easy-to-interpret*) and 124 *high-BD* NCs (62 *difficult-* and 62 *easy-to-interpret*).

### Radiomic Analysis

#### Extraction of Global Radiomic Features

**Fig. 1:**
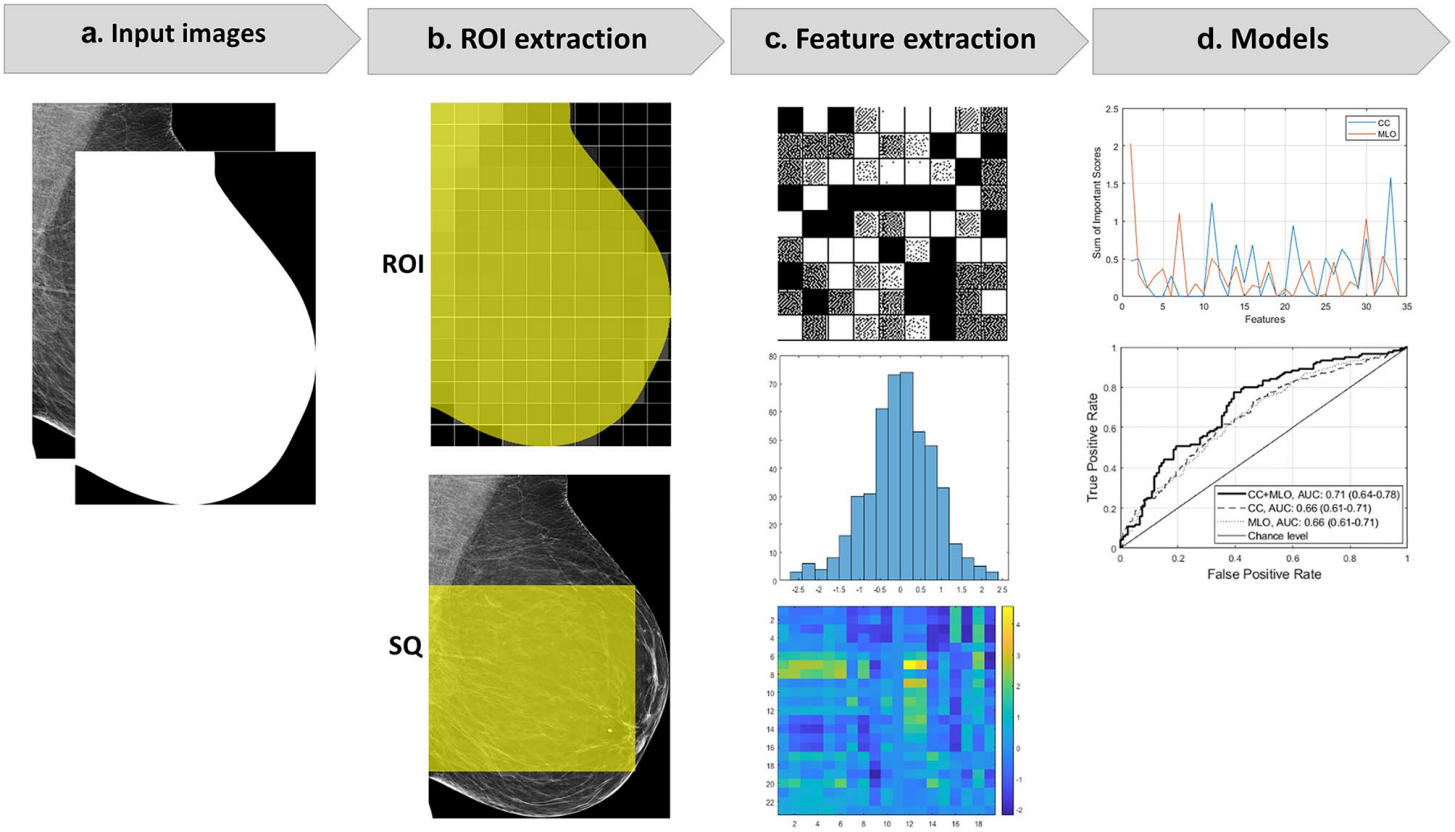
Study workflow. First, images and masks were acquired and used as input images (**a**). Using the lattice- (ROI) and squared-based (SQ) approaches (**b**), 34 global radiomic features (GRFs) per image were then extracted from the region of interest (yellow region) and normalised (image gray intensity mean = 0 and standard deviation = 1) (**c**). Lastly, three machine learning models for predicting *difficult-to-interpret* mammographic normal cases were built and evaluated (**d**)

The first step in the radiomics analysis pipeline was to obtain input mammographic images and binary (black and white) masks. In total, 956 mammography DICOM images (4 images per case × 239 NCs: 120 *difficult-* and 119 *easy-to-interpret*) were acquired. Using a standard gray level thresholding value of 100 (all pixel intensities above the threshold value were converted to white while the rest were converted to black), binary masks were generated from 956 DICOM images to extract the breast region from the surrounding image background, removing undesirable labels, markers, and other artifacts. Where necessary, a manual adjustment on the thresholding value was applied to ensure the binary masks were created accurately. Next, for computational efficiency purposes, the DICOM images and masks were converted to a TIFF file format, and all the right CC and MLO views of mammograms were flipped to the left so that all mammograms had consistent chest wall on the left side. The TIFF images and binary masks were then cropped based on the maximum size of breast region and used as input images for the radiomic analysis (Fig. 1).

Extraction of GRFs was performed using the handcrafted features approach [38] based on region of interests defined through two methods: (1) multiple regions of interests defined by the lattice-based approach covering the entire breast image (ROI) [52, 53] and (2) largest square rectangular box inscribed within breast (SQ) [54–56]. Unlike the previous studies’ techniques [52–56], we included the pectoral muscle for our radiomic analysis as many of our cases had FP annotations near the pectoral muscle area.

A total of 34 GRFs per image (gray level co-occurrence matrix/GLCM-based Haralick texture features (*n* = 30) [57, 58], first-order statistics/FOS features (*n* = 2) [33, 58], and neighbourhood gray tone difference matrix/NGTDM texture features (*n* = 2) [58, 59], Table 2) were extracted using our in-house MATLAB programs. These features were selected as they have been shown to be valuable descriptors of mammographic appearances in measuring the contrast values of spatial inter-relationships between neighbouring pixels of the image (GLCM and NGTDM) and the deviation of single pixel intensity value within the image region of interest (FOS) [33, 57–62]. The extracted features were then normalised using a *z*-score algorithm to calibrate image gray intensity mean to 0 and standard deviation values to 1 [63–65]. The ROI features were analysed using MATLAB distinct block processing method with block size 214 × 214 pixels and summarised using the standard deviation approach.

**Table 2:** List of extracted global radiomic features for this study (n = 34)

**No.**	**Name**	***p*-value**	**Importance score from CC model**	**Importance score from MLO model**	**Feature class**	**Parameters**
**1**	ROI_Std_Correlation_3	0.000^a^	0.47	2.03^b,c^	GLCM [57, 58]	3 pixel distance between the pixel of interest and its neighbour
**2**	ROI_Std_Difference_entropy _3	0.169	0.5	0.29		
**3**	ROI_Std_Dissimilarity_3	0.245	0.14	0.11		
**4**	ROI_Std_Difference_variance_3	0.69	0	0.27		
**5**	ROI_Std_Energy_3	0.025^a^	0	0.37		
**6**	ROI_Std_Entropy_3	0.022^a^	0.28	0		
**7**	ROI_Std_Homogeneity_3	0.343	0	1.10^b^		
**8**	ROI_Std_Information_measure_of_correlation1 _3	0.87	0	0		
**9**	ROI_Std_Information_measure_of_correlation2 _3	0.582	0	0.17		
**10**	ROI_Std_Maximum_probability_3	0.029^a^	0	0.03		
**11**	ROI_Std_Sum_entropy_3	0.000^a^	1.25^b^	0.5		
**12**	ROI_Std_Sum_variance_3	0.649	0.25	0.36		
**13**	ROI_Std_Coarseness_3	0.945	0	0.13	NGTDM [58, 59]	3 × 3 neighbourhood size
**14**	ROI_Std_Cluster_prominence_3	0.398	0.69	0.4	GLCM [57, 58]	3 pixel distance between the pixel of interest and its neighbour
**15**	ROI_Std_Cluster_shade_3	0.373	0.18	0		
**16**	ROI_Std_Range_all	0.006^a^	0.68	0.15	FOS [33, 58]	n/a
**17**	ROI_Std_Sum_of_squares_variance_9	0.215	0	0.12	GLCM [57, 58]	9 pixel distance between the pixel of interest and its neighbour
**18**	SQ_Correlation_9	0.006^a^	0.31	0.46		
**19**	SQ_Difference_entropy _9	0.000^a^	0	0		
**20**	SQ_Dissimilarity_9	0.000^s^	0.05	0.11		
**21**	SQ_Difference_variance_9	0.000^a^	0.94^b^	0		
**22**	SQ_Energy_9	0.000^a^	0.33	0.28		
**23**	SQ_Entropy_9	0.000^a^	0.07	0.47		
**24**	SQ_Homogeneity_9	0.000^a^	0	0		
**25**	SQ_Information_measure_of_correlation1 _9	0.000^a^	0.51	0.03		
**26**	SQ_Information_measure_of_correlation2 _9	0.000^a^	0.3	0.46		
**27**	SQ_Maximum_probability_9	0.000^a^	0.63	0		
**28**	SQ_Sum_entropy_9	0.001^a^	0.48	0.19		
**29**	SQ_Sum_variance_9	0.000^a^	0.11	0.13		
**30**	SQ_Coarseness_9	0.535	0.77	1.03^b^	NGTDM [58, 59]	9 × 9 neighbourhood size
**31**	SQ_Cluster_prominence_9	0.511	0.02	0	GLCM [57, 58]	9 pixel distance between the pixel of interest and its neighbour
**32**	SQ_Cluster_shade_9	0.182	0.22	0.54		
**33**	SQ_Range_all	0.000^a^	1.57^b,c^	0.31	FOS [33, 58]	n/a
**34**	SQ_Sum_of_squares_variance_9	0.000^a^	0	0	GLCM [57, 58]	9 pixel distance between the pixel of interest and its neighbour

*CC* craniocaudal, *FOS *first order statistics, *GLCM *gray level co-occurrence matrix, *MLO* mediolateral oblique, *NGTDM* neighbourhood gray tone difference matrix, *n/a* not applicable, *ROI* multiple regions of interests defined by the lattice-based approach covering the entire breast image,* SQ* largest square rectangular box inscribed within breast, *Std* standard deviation

^a^20 features with statistically significant difference between *difficult-* and *easy-to-interpret* normal cases, *p*-value < 0.05

^b^Top 6 and ^c^top 2 useful features from the machine learning CC/MLO models. The higher the value, the more useful the features were for the predictive models

#### Model Building

Bivariate analysis comparing potential GRFs to the *difficult-* and *easy-to-interpret* NCs was conducted.

**Fig. 2:**
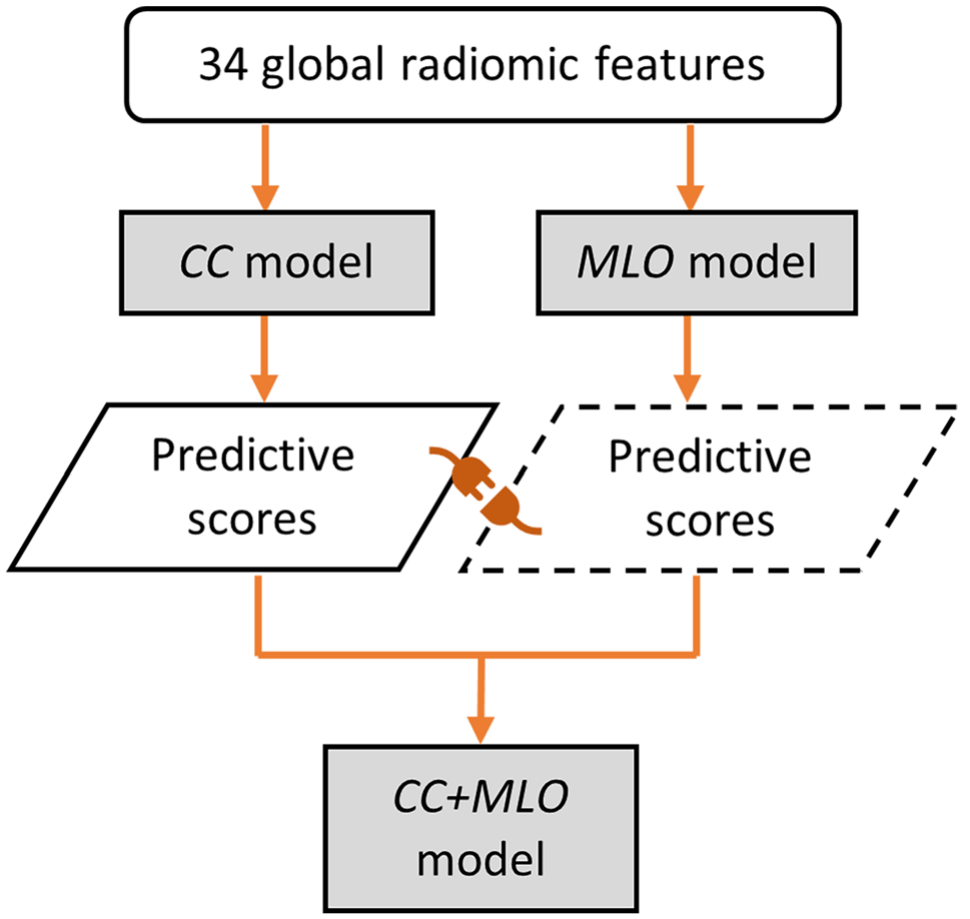
Model building. First, the *CC* and *MLO* model were built using the 34 global radiomic features extracted from the responding CC and MLO view images only. The *CC* + *MLO* model was the median predictive scores of the *CC* and *MLO* models. CC = craniocaudal, MLO = mediolateral oblique

To predict *difficult-* from *easy-to-interpret* NCs, three binary machine learning (ML) classification models were built: (1) *CC*, (2) *MLO*, and (3) *CC* + *MLO* model. Firstly, in constructing the *CC* and *MLO* classification models, the 34 GRFs, obtained from the corresponding right and left CC and MLO view images only, were fed into MATLAB ensemble of 500 decision trees (i.e., random forest) enhanced with LogitBoost ensemble aggregation algorithm (i.e., adaptive logistic regression) [66–68]. We selected the random forest technique because a random feature selection is embedded within the model which can reduce the features overfitting problem while producing interpretable models with automatic estimation of feature importance [68]. To recognise useful GRFs for the predictive *CC* and *MLO* models, importance scores for each feature were computed through a feature importance analysis using MATLAB’s predictor importance algorithm. Importance scores indicate how useful each feature was in the construction of the decision trees within the model. The higher the value, the more useful the features were in making the prediction for the model. Finally, in building the *CC* + *MLO* model, we utilized a late fusion method [69] which consisted of taking the median predictive scores from the constructed *CC* and *MLO* models (Fig. 2).

### Statistical Analysis and Validation

To assess the performance of the models on our dataset, we needed to measure how well the predictions made by the model matched the observed data. Thus, we trained and validated the *CC* and *MLO* models using the leave-one-out-cross-validation approach, a reliable, unbiased, and accurate validation method for evaluating the performance of a machine learning model, assuring no data leakage from the training to the testing phase [62, 70]. This means that each time the model was trained, one case (all images belonging to that case) was left out and used once as a test set to validate the predictive performance of the model, while the remaining cases were used to train the model. This process was repeated for all cases until each case was left out once. The performances of the three models for differentiating *difficult-* from *easy-to-interpret* NCs were evaluated by the area under receiver operating characteristic curve (AUC). A DeLong test [71, 72] was used to determine if there was a statistically significant difference in the performance between the three models (*CC* vs *CC* + *MLO*, *MLO* vs *CC* + *MLO*, and *CC* vs *MLO* model*)*. A two-sided *p* value of < 0.05 was used as the criterion of statistically significant difference.

A scree test of exploratory factor analysis [73] was used to determine useful GRFs based on their total importance scores from the *CC* and *MLO* models.

Using a Kruskal–Wallis test, we explored if the 34 GRFs differed between *difficult-* vs *easy-to-interpret* NCs and if the median difficulty level differed among *low-* vs *high-BD* NCs (*difficult-* and *easy-to-interpret*). A *p* value of < 0.05 was considered statistically significant.

All radiomics analysis pipeline and statistical analysis were conducted using MATLAB R2021a (MathWorks, Natick, MA, USA) apart from the DeLong test which was performed using pROC package [74] in RStudio 2021.09.0.351 (RStudio Team, Boston, MA, USA)/R 4.1.2 (R Core Team, Vienna, Austria).

## Results

### Radiomics Predicting Difficult Normal Cases

**Fig. 3:**
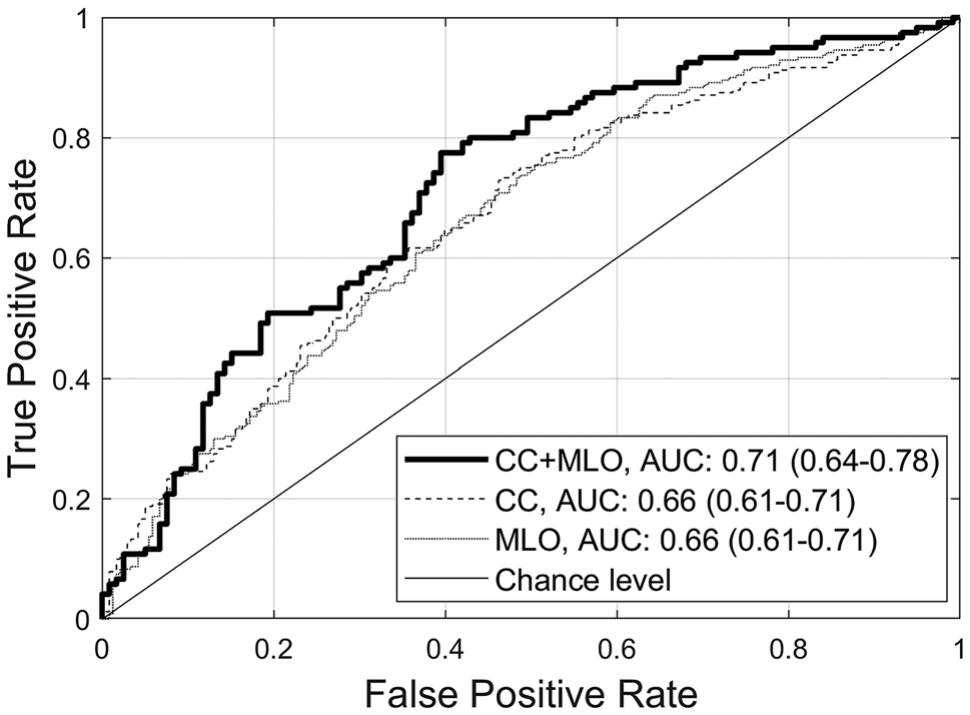
Area under the receiver operating characteristic curve (AUC) for three classifiers. When differentiating *difficult-* from *easy-to-interpret* normal cases, using GRFs from CC or MLO view images only, the performance of *CC* and *MLO* models achieved similar AUCs of 0.66 (95% CI: 0.61–0.71). However, when fusing GRFs from both CC and MLO images using the median predictive scores from both *CC* and *MLO* models, the combined *CC* + *MLO* model yielded a higher AUC of 0.71 (95% CI: 0.64–0.78). CC = craniocaudal, CI = confidence interval, MLO = mediolateral oblique

Figure 3 demonstrates the comparisons of the overall classification performance. When only GRFs from either CC or MLO views were included, corresponding *CC* and *MLO* models achieved similar 0.66 AUCs (95% confidence interval/CI: 0.61–0.71). However, when GRFs from both CC and MLO views were combined using the median predictive scores from both *CC* and *MLO* models, the *CC* + *MLO* model reached a higher AUC value of 0.71 (95% CI: 0.64–0.78). Nevertheless, the DeLong test shows that there was no statistically significant difference in the performance between the three models (*Z* =  − 1.561, *p* = 0.119, *CC* vs *CC* + *MLO*; *Z* =  − 1.846, *p* = 0.065, *MLO* vs *CC* + *MLO*; and *Z* = 0.198, *p* = 0.843, *CC* vs *MLO* model).

### Significant Global Radiomic Features

**Fig. 4:**
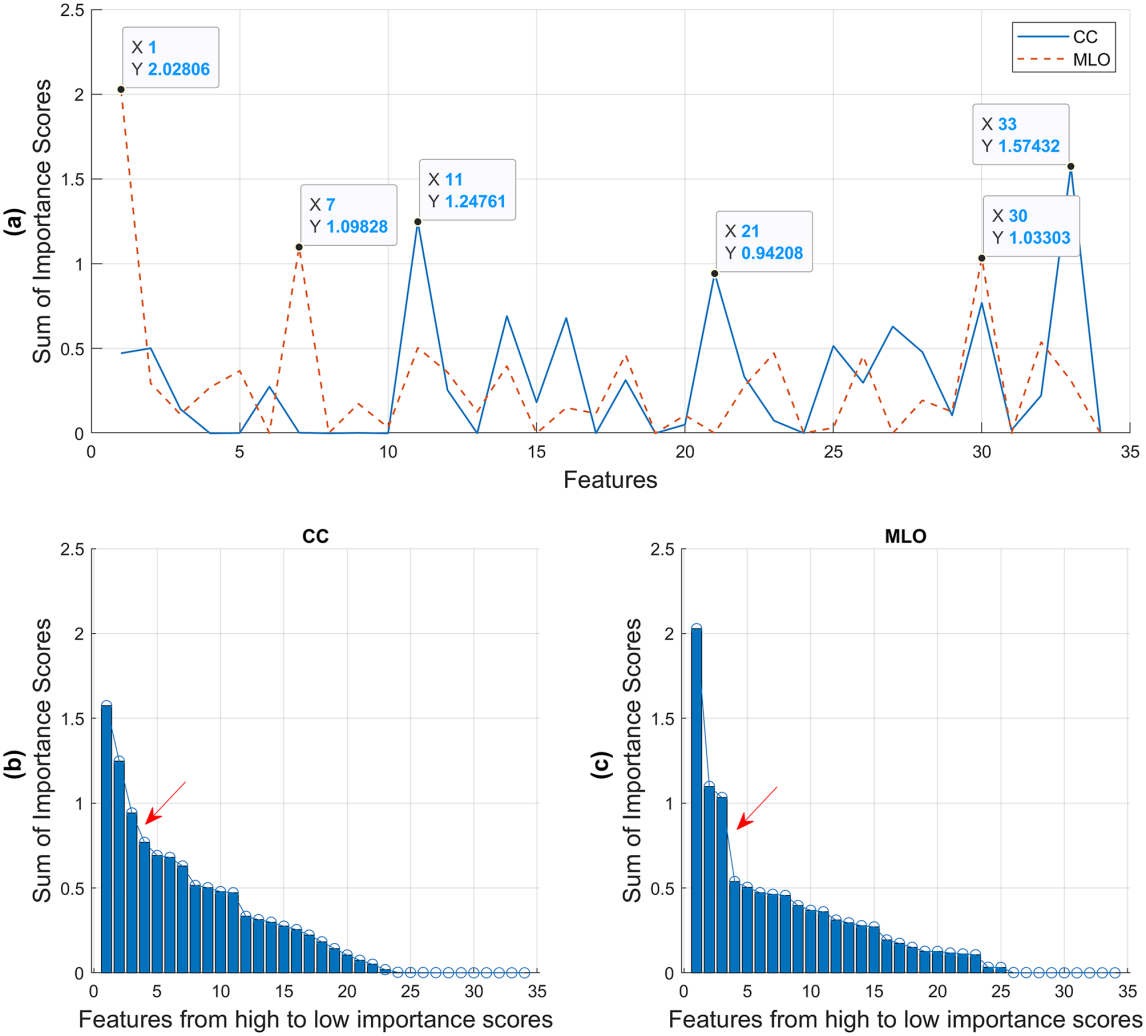
Significant global radiomic features based on their importance scores derived from the predictive *CC* and *MLO* models.** a** Among the 34 global radiomic features, three features with highest importance scores from *CC* model were *ROI_Std_Sum_entropy_3* (features no. 11) and *SQ_Difference_variance_9* (features no. 21) of GLCM, and *SQ_Range_all* (features no. 33) of FOS, determined based on **b** the scree plot indicating the steep slope occurred after the third highest features (shown by the red arrow)*.* Similarly, three highest importance score features from *MLO* model were *ROI_Std_Correlation_3* (features no. 1) and *ROI_Std_Homogeneity_3* (features no. 7) of GLCM, and *SQ_Coarseness_9* (features no. 30) of NGTDM according to **c** the scree plot with the red arrow showing the steep slope occurred after the third highest features. CC = craniocaudal, FOS = first order statistics, GLCM = gray level co-occurrence matrix, MLO = mediolateral oblique, NGTDM = neighbourhood gray tone difference matrix, ROI = multiple regions of interests defined by the lattice-based approach covering the entire breast image, SQ = largest square rectangular box inscribed within breast, Std = standard deviation

Among the 34 extracted GRFs, 20 features (Table 2) showed a statistically significant difference between *difficult-* vs *easy-to-interpret* NCs (*p* < 0.05). When the combined 34 features were used for the ML predictive models, six significant features from predictive *CC* and *MLO* models demonstrated highest importance scores when compared between the 34 features based on the scree test (Fig. 4). The three most important features from *CC* model were *ROI_Std_Sum_entropy_3*, *SQ_Difference_variance_9*, and *SQ_Range_all* with mean importance score of 1.25*.* The three most important features from *MLO* model were *ROI_Std_Correlation_3*, *ROI_Std_Homogeneity_3*, and *SQ_Coarseness_9* including 1.39 mean importance score (Table 3).

**Table 3:** List of important global radiomic features based on the machine learning models

**Feature no.**	**Name**	**Models**	**Feature class**	**Importance scores**	**Mean scores**	**Description**
**11**	ROI_Std_Sum_entropy_3	CC	GLCM [57, 58]	1.25	1.25	Describing the sum of randomness in the gray level distribution of the image
**21**	SQ_Difference_variance_9	CC	GLCM [57, 58]	0.94		Measuring heterogeneity that places higher weights on differing intensity level pairs that deviate more from the mean
**33**	SQ_Range_all	CC	FOS [58, 59]	1.57^a^		Describing the difference between maximum and minimum of image gray level values
**1**	ROI_Std_Correlation_3	MLO	GLCM [57, 58]	2.03^a^	1.39	Measuring similarity or linear dependency of gray level values between the two neighbouring pixels and representing the image’s smoothing gradient of texture pattern
**7**	ROI_Std_Homogeneity_3	MLO	GLCM [57, 58]	1.10		Measuring the closeness of the distribution of elements in the GLCM to the GLCM diagonal
**30**	SQ_Coarseness_9	MLO	NGTDM [58, 59]	1.03		Measuring average difference between the center pixel and its neighbourhood and is an indication of the spatial rate of change

*CC* craniocaudal, *FOS *first order statistics, *GLCM* gray level co-occurrence matrix, *MLO* mediolateral oblique, *NGTDM* neighbourhood gray tone difference matrix, *ROI* multiple regions of interests defined by the lattice-based approach covering the entire breast image, *SQ* largest square rectangular box inscribed within breast, *Std* standard deviation

^a^Features with highest importance score based on the *CC* / *MLO* models

### Breast Density of Difficult Normal Cases

**Fig. 5:**
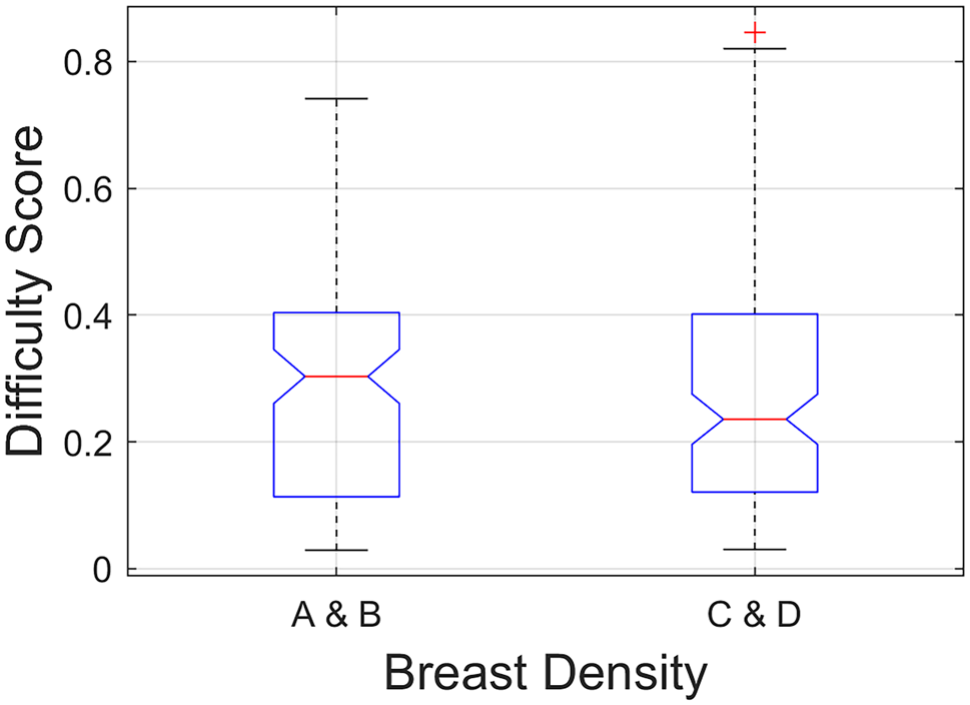
Comparison of difficulty level between ***low-BD*** (A&B) vs ***high-BD*** (C&D) normal cases**.** No statistically significant difference in the difficulty level was found between the two groups (*H*(1) = 0.14, *p* = 0.709). A = Fatty: 0–25% glandular, B = Fibroglandular: 25–50% glandular, BD = breast density, C = Heterogenous: 51–75% glandular, D = Extremely dense: 76–100% glandular

Figure 5 illustrates the comparison of the traditional BD-based difficulty level between *low-* vs *high-BD* NCs (*difficult-* and *easy-to-interpret*). The result from the Kruskal–Wallis test showed no statistically significant difference in the difficulty level between the two groups (*H*(1) = 0.14, *p* = 0.709).

## Discussion

This first exploratory study investigated if by employing a radiomic approach to describe the global mammographic features, *difficult-to-interpret* NCs can be predicted. Our work based on the three ML models (*CC*, *MLO*, and *CC* + *MLO*) exhibited that GRFs had the ability to accurately differentiate *difficult-* from *easy-to-interpret* NCs (AUCs up to 0.71). A few earlier studies indicated the value of local radiomic features from specific tumor/FP regions in predicting radiology trainees’ error making patterns (AUCs up to 0.61) [39, 40]. However, we focused on GRFs’ efficacy in predicting *difficult-to-interpret* NCs (i.e., an objective measure of difficulty based on readers’ error rate [31]) based on the significance of radiomics in describing mammographic characteristics [38] and global image processing (i.e., global gist/radiological first impression) in diagnostic decision making [41–44]. Furthermore, our combined *CC* + *MLO* model particularly integrating features from both CC and MLO views of mammograms (using the median predictive scores from both *CC* and *MLO* models) showed higher performance (0.71 AUC, 0.64–0.78) when compared to *CC* or *MLO* model alone (0.66 AUCs, 0.61–0.71) using only features from corresponding CC or MLO images (Fig. 3). This finding suggests that GRFs from both CC and MLO views of mammographic images can provide complementary useful information in predicting *difficult-to-interpret* NCs. Nonetheless, more and larger future works are required to confirm this finding. While the results of comparing *CC* to *MLO* model showed a clearer indication of no statistically significant difference between the model’s performance (*Z* = 0.198, *p* = 0.843), the comparisons of *CC* and *MLO* to the combined *CC* + *MLO* model were indefinite (*Z* =  − 1.561, *p* = 0.119 and *Z* =  − 1.846, *p* = 0.065, respectively) which suggests more conclusive answers might need a larger sample.

Previous studies reported that the global gist signal without any localised target information could be used to classify normal from abnormal mammograms [41, 42]. An earlier study [75] highlighted the importance of the global gist signal in guiding a visual search and making a diagnosis decision when interpreting difficult cancer cases (unreported retrospectively identified cancers on mammograms). Thus, we hypothesised that *difficult-to-interpret* NCs have global image characteristics which may resemble an abnormal appearance and differ from *easy-to-interpret* images. If a difficult normal image seems to be abnormal at first, this often results in prolonged interpretations, and subsequently, the chance of FP errors may increase since FP errors are often reported at the later stages of prolonged visual searches on normal mammograms [76].

Our work suggests that the GRFs (describing the entire image and largest square rectangular inscribed within breast) contained within mammographic images can help to identify *difficult-to-interpret* NCs. To date the process of assessing difficulty, NCs tends to depend heavily on a manual and subjective evaluation of radiological experts with high-BD cases often perceived as more difficult [19, 26–28, 30]. However, similar to previous studies [28, 29], our finding (Fig. 5) demonstrated no statistically significant difference (*H*(1) = 0.14, *p* = 0.709) observed in the difficulty level based on the traditional BD class between *low-* and *high-BD* NCs, suggesting that BD classifications may not be suitable for estimating difficult NCs. Conversely, our GRFs can provide important details for recognising mammographic features of difficult NCs that could be used to facilitate an automatic and consistent approach for evaluating difficult NCs, building more predictable test sets and consistent educational materials for radiologists [14–16, 21]. It can also facilitate a reliable and effective objective measurement of the recall rate performance of each reader as well as screening program over time given that readers’ performance in test set based education can correspond well with their clinical performance [15, 20, 22–25].

Additionally, our approach may be used as an augmented clinical artificial intelligence (AI) tool to notify clinical managers about *difficult-to-interpret* NCs so that more experienced readers can be assigned to the case or an appropriate strategy in pairing readers could be employed to optimise diagnostic accuracy. A single reading strategy, instead of a double reading, can also be exploited for *easy-to-interpret* NCs, reducing readers’ workload since NCs constitute about 99.5% of cases in screening programs [13, 77–79]. Nonetheless, more work is required to further explore and verify such an AI tool’s effectiveness and reliability before incorporating it into a clinical practice.

Our ML approach based on GRFs indicated that the overall mammographic appearance is an important factor in analysing *difficult-to-interpret* NCs. From the total of 34 features, 20 features showed statistically significant difference between *difficult-* vs *easy-to-interpret* NCs (*p* < 0.05, Table 2). Nevertheless, when the combined 34 features were used for the ML-based predictive models, more redundant features were discovered, resulted in six helpful GRFs in describing *difficult-to-interpret* NCs based on their importance scores (Fig. 4 and Table 3). The MLO- rather than the CC-derived parameters appeared to be more valuable which may be linked to the MLO-based GRFs involving more breast tissue including the pectoral muscle. This finding, though, needs further investigation by larger future studies.

Some limitations of this study should be acknowledged. The prevalence of NCs in the BREAST test sets used in this study was lower than the real clinical practice. Also, this first proof-of-concept study only examined 34 mammography-based handcrafted GRFs’ ability in predicting *difficult-to-interpret* NCs based on the average difficulty scores of all readers. Moreover, determining important GRFs based on the scree test [73] (Fig. 4b, c) was less clear cut for the *CC* model when compared to the *MLO*. Future works should explore other potential useful CC-based GRFs (e.g., feature no. 30, 14, 16, and 27) and type of radiomic features (e.g., local handcrafted or deep learning features) and using equipment from other imaging vendors (e.g., Canon, Canon Medical Systems Corporation, Otawara, Tochigi, Japan). Finally, separate models for predicting difficult cases of readers at various experience levels should be investigated.

## Conclusions

Our findings suggested that quantitative GRFs extracted from mammograms are helpful for accurately distinguishing *difficult-* from *easy-to-interpret* NCs, denoting the importance of global gist in making a diagnostic decision. Six important features from the classification models were recognised and highlighted. Our findings could be useful for radiology educations and mammography screening programs in creating more predictable test sets, and reliable and effective objective measurement of the recall rate performance of each reader and screening program over time, as well as optimising single and double reading practice.

## Data Availability

The data that support the findings of this study are available from BREAST but restrictions apply to the availability of these data, which were used under license for the current study, and so are not publicly available.Data are however available from the authors upon reasonable request and with permission of BREAST.

## Abbreviations

AI: Artificial intelligence
AUC: Area under receiver operating characteristic curve
BD: Breast density
BI-RADS: Breast Imaging and Reporting Data System
BREAST: BreastScreen Reader Assessment Strategy
CC: Craniocaudal
CI: Confidence interval
DICOM: Digital Imaging and Communications in Medicine
FOS: First order statistics
FP: False positive
GLCM: Gray level co-occurrence matrix
GRFs: Global radiomic features
ML: Machine Learning
MLO: Mediolateral oblique
NCs: Normal cases
NGTDM: Neighbourhood gray tone difference matrix
RANZCR: Royal Australian and New Zealand College of Radiologists
ROI: Multiple regions of interests defined by the lattice-based approach covering the entire breast image
SQ: Largest square rectangular box inscribed within breast
